# Supplementary material for: Bayesian refinement of protein structures and ensembles against SAXS data using molecular dynamics
Source: PLoS Comput Biol. 2017 Oct 18;13(10):e1005800. doi: 10.1371/journal.pcbi.1005800 (PMC5662244; doi:10.1371/journal.pcbi.1005800)
Supplement: S1 Archive — (BZ2) [file pcbi.1005800.s011.tar.bz2 › gromacs_4.62_bayesian_saxs_refi/share/html/online/m2p.html]

m2p file format


## m2p file format

|  |  |
| --- | --- |
| Main Table of Contents | **VERSION 4.6** |
| GROMACS homepage | **Sat 19 Jan 2013** |

---

### Description

The m2p file format contains input options for the
xpm2ps program. All of these options
are very easy to comprehend when you look at the PosScript(tm) output
from xpm2ps.

```
; Command line options of xpm2ps override the parameters in this file
black&white              = no           ; Obsolete
titlefont                = Times-Roman  ; A PostScript Font
titlefontsize            = 20           ; Font size (pt)
legend                   = yes          ; Show the legend
legendfont               = Times-Roman  ; A PostScript Font
legendlabel              =              ; Used when there is none in the .xpm
legend2label             =              ; Used when merging two xpm's 
legendfontsize           = 14           ; Font size (pt)
xbox                     = 2.0          ; x-size of a matrix element
ybox                     = 2.0          ; y-size of a matrix element
matrixspacing            = 20.0         ; Space between 2 matrices
xoffset                  = 0.0          ; Between matrix and bounding box
yoffset                  = 0.0          ; Between matrix and bounding box
x-major                  = 20           ; Major ticks on x axis every .. frames
x-minor                  = 5            ; Id. Minor ticks
x-firstmajor             = 0            ; First frame for major tick
x-majorat0               = no           ; Major tick at first frame
x-majorticklen           = 8.0          ; x-majorticklength
x-minorticklen           = 4.0          ; x-minorticklength
x-label                  =              ; Used when there is none in the .xpm
x-fontsize               = 16           ; Font size (pt)
x-font                   = Times-Roman  ; A PostScript Font 
x-tickfontsize           = 10           ; Font size (pt)
x-tickfont               = Helvetica    ; A PostScript Font
y-major                  = 20
y-minor                  = 5
y-firstmajor             = 0
y-majorat0               = no
y-majorticklen           = 8.0
y-minorticklen           = 4.0
y-label                  = 
y-fontsize               = 16
y-font                   = Times-Roman
y-tickfontsize           = 10
y-tickfont               = Helvetica
```

---

http://www.gromacs.org
